# 

**Published:** 1891-04

**Authors:** 


### LITERARY.

THE MACKAY STAR MAP.—During the years 1887–8, the editor of the *Esoteric* wrote a series of 21 articles, entitled "Naked Eye Astronomy," which became very popular, and did much to open the eyes of the readers of that journal to the beauties of the oldest and mightiest of sciences. This map before us is an outcome of these popular essays, and constitutes a most valuable and instructive feature of popular education, in fields least understood of all, by the masses.—Esoteric Co., Boston, price 25 cts.

CRUDE AND INFINITESIMAL DOSES, by Henry Sheffield, M. D., Nashville, Tenn., is a very forcible and philosophical exposition of the Homeopathic school of medicine, as inaugurated by the renowned Hahnemann, and practised successfully by thousands of his adherents in this country, with a comparison of the two leading systems of administering drugs, and an affirmation of *the less of them the better*, which fully accords with the views of the reviewer.

AM I JEW OR GENTILE? OR THE GENEALOGY OF JESUS CHRIST, ASSERTING HIS DIVINITY.—By Thomas A. Davies, author of a number of kindred works. A unique and curious book, pointing out alleged errors in reference to this most important subject, worthy the attention of the Theologian and the general reader. Published and for sale by E. H. Coffin, 49 John street, New York. Price 25 cents.

HYGIENE OF MOTHERHOOD,—No. 6, of Dr. John Sheppman's articles under the above title, arrived after we had gone to press. It will appear in our May issue. The Doctor complains of some omissions, which in our haste, occurred in part 5th. We confess the fault was with this office.

OUR MAGAZINE EXCHANGES are not numerous, but they are admirable in quality. Among them are

THE CENTURY; LEND A HAND; BELFORD'S; HOME MAKER; THE MONIST; VICK'S; THE HORTICULTURAL; OUR LITTLE MEN AND WOMEN, and BABYLAND. If there are any better of their several classes, we are not aware of it.

Diabetes. Its Causes, Symptoms and Treatment, by Charles W. Purdy, M. D., author of "Bright's Disease," etc., etc., with Clinical Illustrations, and rules of diet for diabetic subjects.

Diabetes is a disorder that gives little warning of its approach and the patient frequently finds himself in its toils without discovering its warnings. The extended treatise of Dr. Purdy goes thoroughly into his subject, carefully tracing the history, symptoms, and most approved methods of treating this disease, so often fatal to both young and old. We learn too, that it is hereditary, sometimes manifesting itself in tender infancy, and common to both sexes, with a prevalence for the male of about two to one. Its classification and clinical consideration will be found of great value to the medical practitioner.

F. A. Davis, Publisher, Philadelphia, pp. 184, price $1.25 net.

The Daughter; Her Health, Education and Wedlock. By Wm. M. Capp, M. D.

This is a handsome little volume of 144 pp., printed and bound in the highest style of the art.

Its title expresses the three leading features in a young woman's life, and their treatment embraces much useful, nay, indispensable, information. The book takes a wider range than its title indicates, and deals with the home life employments, wifehood and motherhood of women, in sickness and in health, in a practical common sense manner, calculated to impart much valuable information and benefit all classes of readers. It recognizes the differences in organisms, and their needs, by stating that "no one kind of prepared food will suit all infants." a fact too frequently overlooked. Of medicines, the author says, "Medicines like crutches, should be used only as temporary expedients to help in certain difficulties." Amen, to this say we.

F. A. Davis, Publisher, Philadelphia. Price, net, $1.00.

A Study of Sterility. Its Causes and Treatment. By Thomas W. Kay, M. D., Scranton, Pa.

This valuable treatise of 50 pp. was a first prize essay of the Alumni Association of the College of Physicians and Surgeons, Baltimore. The prize was evidently worthily bestowed.

Cornell University, College of Agriculture. Third Annual Report, 1890. Illustrated, 54 pp.

All question of the practical value of this department would be set at rest by a careful perusal of this excellent report.

Herba Vita.—We are constantly receiving testimonials of the wonderful curative properties of this simple and harmless remedy, for nearly all the diseases common to this country, particularly in the "new south" and mighty west

*It is not a tea*, but a combination of rare imported roots and herbs grown in Egypt and other sunny lands, and operates with magical effect upon disordered systems. It will both prevent and cure disease. Try it.

THE AMESBURY SOUVENIR.—We have received a souvenir edition of the *Amesbury Daily*, printed in large magazine form, with elegant lithograph designs upon the covers, and profusely illustrated with admirable half-tone cuts, including nearly one hundred portraits of prominent citizens and business men of Amesbury, a town well known as the residence of the poet Whittier, and the birthplace of the veteran editor of the *Banner of Light*, Luther Colby.

Amesbury is a live, progressive place, noted for its extensive carriage industry. The typographical and artistic appearance of the book reflects great credit upon the Amesbury Publishing Co., consisting of J. M. & I. J. Potter, publishers of the *Yankee Blade*, *New England Fireside*, *New England Magazine* and *Amesbury Vehicle*. The town is the carriage centre of America and there are few on the continent more enterprising, and deservedly prosperous.

THE SOUL OF MAN.—Under this title Dr. Paul Carus, the brilliant editor of *The Open Court*, has collected, arranged and elaborated many of the able physiological, psychological, neurological and sociological articles with which his name has become identified, the whole forming an interesting and instructive study of that most subtle of subjects—the human mind—discussed from the position of a positive monist, as clearly expressed in the preface. As an exposition of "scientific religion," this work is direct and forcible; as a source of information it is extensive and accurate. A pioneer in the literature of comprehensive experimental psychology, Dr. Carus is destined to arouse a host of controversialists, among whom he will certainly hold his own.

"The Soul of Man" is published in a handsome 8vo vol. of 480 pp., with 152 explanatory illustrations and diagrams, by The Open Court Pub. Co. of Chicago, Ill. Price $3.00.

